# Host genomics in diagnosing inborn errors of immunity and tailoring infectious disease treatments

**DOI:** 10.3389/fgene.2026.1984349

**Published:** 2026-10-28

**Authors:** Lucas Buyon, Jonathan S. Berg, Dusan Bogunovic, Eric D. Green, Steven M. Holland, Jahnavi Narula, Sarah W. Read, Alauna Rupert, Jeanne M. Marrazzo, Jennifer M. Puck

**Affiliations:** 1 Formerly of National Institute of Allergy and Infectious Diseases, National Institutes of Health, Bethesda, MD, United States; 2 University of North Carolina School of Medicine, Chapel Hill, NC, United States; 3 Columbia University Medical Center, New York, NY, United States; 4 Formerly of National Human Genome Research Institute, National Institutes of Health, Bethesda, MD, United States; 5 National Institute of Allergy and Infectious Diseases, National Institutes of Health, Bethesda, MD, United States; 6 University of Queensland School of Medicine, Brisbane, QLD, Australia; 7 University of Michigan, Ann Arbor, MI, United States; 8 Infectious Disease Society of America, Arlington, VA, United States; 9 School of Medicine, University of California San Francisco, San Francisco, CA, United States

**Keywords:** clinical decision support, disease risk stratification, electronic health record (EHR), genomic variant curation, inborn error of immunity (IEI), infectious diseases, precision medicine, primary immunodeficiency

## Abstract

Genomic technologies are widely applied clinically to identify pathogens and their antimicrobial susceptibilities, but less use is made of patients’ germline genomic variations in infectious disease management. Not only can inborn errors of immunity (IEIs), most of which are rare, be detected before severe complications develop, but host genomics can assist with risk stratification for severe common infections, antimicrobial therapy selection, and pharmacogenomic-guided dosing. Over 30 clinical immunologists, infectious disease specialists and geneticists met to outline challenges and opportunities concerning host genomics and infectious diseases. Obstacles identified included payer requirements for extensive conventional diagnostics prior to genetic testing, lack of electronic health record (EHR) interoperability, and limited evidence of efficacy and cost-effectiveness given the individual rarity of most IEIs. Priorities to broaden the use of host genomics include publicizing indicators of IEIs, building them into EHR informatics systems, and promoting multidisciplinary approaches to identifying patients with IEIs.

## Introduction

Genomic technologies have ushered in a new era of precision for both clinical medicine and public health (McCarthy et al., 2013). However, while genomics is widely applied in public health research within the fields of infection and immunity, its clinical applications have developed more slowly (Armstrong et al., 2019). In recent years, the successful use of genomic medicine to diagnose and treat heritable immune disorders has highlighted the potential for broader clinical adoption. By leveraging next-generation DNA sequencing (NGS), bioinformatics, and robust data-sharing frameworks, clinicians and researchers can now identify previously unsuspected immune deficiencies and tailor treatment strategies for infectious diseases based on a patient’s unique genomic profile (Akalu and Bogunovic, 2024; Kingsmore et al., 2024). Among the most significant beneficiaries of these advances are individuals with inborn errors of immunity (IEIs) (Akalu and Bogunovic, 2024). In the past, IEIs often went undetected until patients presented with repeated or severe infections, sometimes after suffering irreversible organ damage or life-threatening complications (Akalu and Bogunovic, 2024). Today, genomic analyses can yield diagnoses prior to or early in the disease course, thereby improving therapeutic outcomes and in some cases making possible targeted interventions such as hematopoietic stem cell transplantation or gene therapy (Akalu and Bogunovic, 2024).

Host genomics also offers insights beyond IEIs, including risk stratification for severe common infections, antimicrobial therapy selection, and pharmacogenomic-guided dosing (Kwok et al., 2021). To explore advances and challenges, the National Human Genome Research Institute (NHGRI) and the National Institute of Allergy and Infectious Diseases (NIAID) convened a group of clinical immunologists, infectious disease specialists, geneticists, bioinformaticians, policymakers, and payers to examine current capabilities, challenges, and pressing questions in this area. This manuscript summarizes the NHGRI-NIAID meeting, reviews existing methods for applying host genomic data in the management of IEIs and infectious diseases, outlines obstacles and potential solutions for clinical integration, and identifies priorities to broaden the use of host genomics in medicine. Details of diagnostic techniques and practice guidelines in immunology are beyond the scope of this paper, but the interested reader is referred to the 2026 Joint Task Force on Practice Parameters publication (Orange et al., 2026).

## Detection and treatment of inborn errors of immunity

Genomic medicine’s value is illustrated by its application to improving the detection and care of individuals with IEIs (Akalu and Bogunovic, 2024; Lee and Abraham, 2021). These genetically driven primary immune dysregulation disorders encompass a group of over 550 varied conditions that compromise immune function. While individually rare, their aggregated prevalence has recently been estimated at 1/1,000 to 1/5,000 (Akalu and Bogunovic, 2024). Historically, many IEIs were recognized only after patients suffered multiple and/or life-threatening infections or inflammatory episodes (Akalu and Bogunovic, 2024). Advances in genomic sequencing offer the potential to change these circumstances dramatically by enabling earlier detection of IEIs, often prior to the onset of symptoms (Akalu and Bogunovic, 2024; Lee and Abraham, 2021).

IEIs can be identified by either “phenotype-first” or “genotype-first” approaches. To date the vast majority have been detected through the former, but the latter have increased in frequency due to effective newborn screening (Currier and Puck, 2021). In the phenotype-first model, clinicians initiate genomic testing in response to clinical flags, such as unusual or recurrent infections or episodes of inflammation or autoimmunity, unexplained failure to thrive in an infant or child, or a family history of immune disease (Akalu and Bogunovic, 2024; Currier and Puck, 2021). Such warning signs suggest a possible underlying IEI that may be identified through a genomics-based investigation. This approach, however, requires an astute clinician as well as a patient phenotype, and is best executed when the patient has continuity of clinical care across healthcare providers. Because the time between clinical episodes can vary and may be long, discontinuity of care is common and frequently limits opportunities for early recognition.

Genotype-first strategies involve upfront genomic screening of apparently healthy individuals to identify pathogenic variants in known IEI-related genes. Current approaches, including enumeration of T cell receptor excision circles, or TRECs, and exome or genome sequencing, are particularly valuable in newborn and pediatric populations (Kingsmore et al., 2024; Currier and Puck, 2021; Engelbrecht et al., 2021). Newborn TREC screening for severe combined immunodeficiency (SCID) has substantially improved survival by permitting hematopoietic stem cell transplantation or gene therapy before clinical illness occurs (Currier and Puck, 2021; Thakar et al., 2023). Extending genotype-first programs to the full spectrum of IEIs, however, faces practical challenges such as public acceptance and consent, the high cost of large-scale sequencing, limitations of variant interpretation, need for more genetic counseling capacity, providing follow-up care for true positives and false positives, and variable insurance reimbursement. Determining whether these barriers are offset by cost savings, reductions in morbidity and mortality, and improved quality of life will require robust multicenter studies to assess the financial and clinical impacts of widespread genomic screening.

Genomic sequencing in undiagnosed diseases has repeatedly been shown to be cost-effective (Kingsmore et al., 2024; Rezapour et al., 2023), although such analyses for IEIs are limited. However, indirect evidence such as high diagnostic yield and clinical impact supports its use in these conditions. Diagnostic yields are generally higher in IEIs than in undiagnosed diseases overall, with an average 38% yield for exome sequencing vs. 23% in a recent meta-analysis of undiagnosed diseases (Vorsteveld et al., 2021; Pandey et al., 2025). Clinical impact can also be substantial; the Jeffrey Modell Foundation’s Genetic Sequencing Program for Primary Immunodeficiency found changes in diagnosis and management following sequencing in 35%–39% of 1,398 patients with confirmed or suspected IEIs (Quinn et al., 2022). And cost-effectiveness has been shown convincingly in newborn screening for severe combined immunodeficiency (SCID) (Quinn et al., 2020).

Another challenge with genomic screening is that many rare, but potentially consequential variants are classified as variants of uncertain significance (VUS) due to limited functional and epidemiologic data (Akalu and Bogunovic, 2024). This is a particular problem among individuals from ancestries underrepresented in current genomic datasets but is frequent across all groups, with over 40% of individuals undergoing genetic testing having at least one VUS (Chen et al., 2023). The frequency and unclear impact of VUSs present significant barriers to implementation of host genomic approaches and are frustrating to clinicians looking for definitive answers. Most often, a VUS is simply a variant about which available information is insufficient to meet standard classification criteria (Richards et al., 2015). As such, clinicians should not overinterpret them as likely disease-causing, nor try to act upon them. Some genetic testing laboratories re-evaluate VUSs periodically; interestingly, this most often leads to reclassification as benign or likely benign (Chen et al., 2023).

Variant reclassification depends on collaborative curation efforts to compile and assess phenotyping, experimental and family data where available, and data generated through techniques such as high throughput functional assays and long read sequencing. Continued and expanded collaborative research programs, such as the Clinical Genome Resource (ClinGen), are needed to advance genomic variant interpretation to improve timely, accurate IEI diagnosis (Akalu and Bogunovic, 2024; Richards et al., 2015; ClinGen Consortium, 2025). Large-scale consortia that link clinical, genomic, and phenotypic datasets can refine gene-disease associations and provide clarity about genomic variants (Nieto-Patlán et al., 2025). Databases like ClinGen and ClinVar have become central to genomic variant curation and classification, and disease- or locus-specific databases can also provide valuable, curated data (Caballero-Oteyza et al., 2024). However, clinicians also require user-friendly interfaces and timely updates, ideally integrated into EHR systems, to maximize efficiency in a patient care setting. Such tools would reduce the time between genomic variant discovery and adjudication of a potential link to pathogenicity.

From a treatment perspective, accurate and early genotype-based identification of IEIs can be transformative. Matching patients with genetically informed therapies (e.g., intravenous immunoglobulins, targeted biologics, specialized prophylactic antimicrobial regimens, or even hematopoietic stem cell transplantation or gene therapy) can drastically reduce morbidity (Kingsmore et al., 2024; Thakar et al., 2023; Kingsmore and Cole, 2022). In SCID, knowing the specific causative gene can inform selection of the ideal donor and conditioning regimen for a stem cell transplant and help predict long-term outcomes. Recently, gene therapy has further expanded the therapeutic options for children with select IEIs by directly correcting the underlying molecular defect (Wijeyesinghe and Chinen, 2025). Gene therapy has progressed to include not only gene addition using lentiviral vectors, but also gene editing to correct a mutated allele or knock down gain-of-function variants. To date successful gene editing has been reported in a variety of IEIs and is evolving rapidly (Wijeyesinghe and Chinen, 2025). Expanded application of genotype-first approaches to additional IEIs could yield similarly improved results if supported by sufficient research, established sequence interpretation algorithms, appropriate payment coverage, and robust data-sharing initiatives.

In addition to its direct clinical benefits, early genetic diagnosis reduces reliance on costly and invasive testing (Kingsmore et al., 2024; Kingsmore and Cole, 2022). Rapid and relatively inexpensive flow cytometry tests are useful to enumerate lymphocyte subsets and assess cellular function and disease activity, while genomic analyses can directly identify monogenic disorders (Similuk et al., 2022). By integrating genomic testing earlier in the clinical course, inborn errors of immunity and related syndromic conditions can be diagnosed more rapidly and economically, therapies can be more precisely targeted, and patients may avoid repeated invasive procedures (Kingsmore et al., 2024).

Genetic testing has its own limitations of course, currently providing diagnoses in only about 30% of IEI cases, though diagnostic yield varies widely by setting (Bucciol et al., 2024). Variable penetrance and expressivity can complicate interpretation of variants known to be pathogenic. Given that an exclusive genetic sequencing approach may lead to overdiagnosis, functional validation of variants should be available and integrated into the diagnostic process. In addition, germline DNA sequence is essentially static throughout the lifespan and sequence itself does not change with infectious challenges, though its conformation and expression can change. Techniques that measure more dynamic changes in response to infection and can increase yield include somatic mutation testing, RNA sequencing (transcriptomics), metabolomic profiling, and long read sequencing (Akalu and Bogunovic, 2024; Ochoa et al., 2025; Chinn et al., 2020).

## Host genomics and treatment of infections

Host genomic variation can impact the trajectory of infectious diseases beyond identifying rare IEIs. Genomic variants influencing immune-related pathways can influence disease severity, progression, and responses to therapy for viral, bacterial, fungal and parasitic infections (Kwok et al., 2021; Lim et al., 2008; Duggal et al., 2013). Allelic variation in the major histocompatibility complex has been associated with HIV disease progression, while variants in *IFNL3* and *IFNL4* confer a greater likelihood of spontaneous hepatitis C virus clearance (Duggal et al., 2013; Vergara et al., 2021). *CCR5* deficiency provides partial protection against HIV infection but may predispose carriers to more severe disease following flavivirus infections such as West Nile virus (Lim et al., 2008). In Polynesian populations, otherwise rare *IFNAR1* variants markedly increase the risk of adverse reactions to live attenuated viral vaccines (Bastard et al., 2022). Pathogenic variants affecting proteins in the type I interferon signaling pathway (*IFNAR1, IFNAR2, TYK2, JAK1, STAT1, STAT2*, and *IRF9*) predispose to severe viral and, to a lesser extent, mycobacterial infections (Meyts and Casanova, 2021). Identification of such variants through a genomics‐first approach could guide vaccine decisions and preventive measures. Finally, *APOL1* risk alleles confer resistance to African trypanosomiasis while simultaneously elevating the risk of kidney disease (Daneshpajouhnejad et al., 2022). These observations illustrate how a single genetic variant can exert opposite clinical effects reflecting historical selective pressures (Figure 1).

**FIGURE 1:**
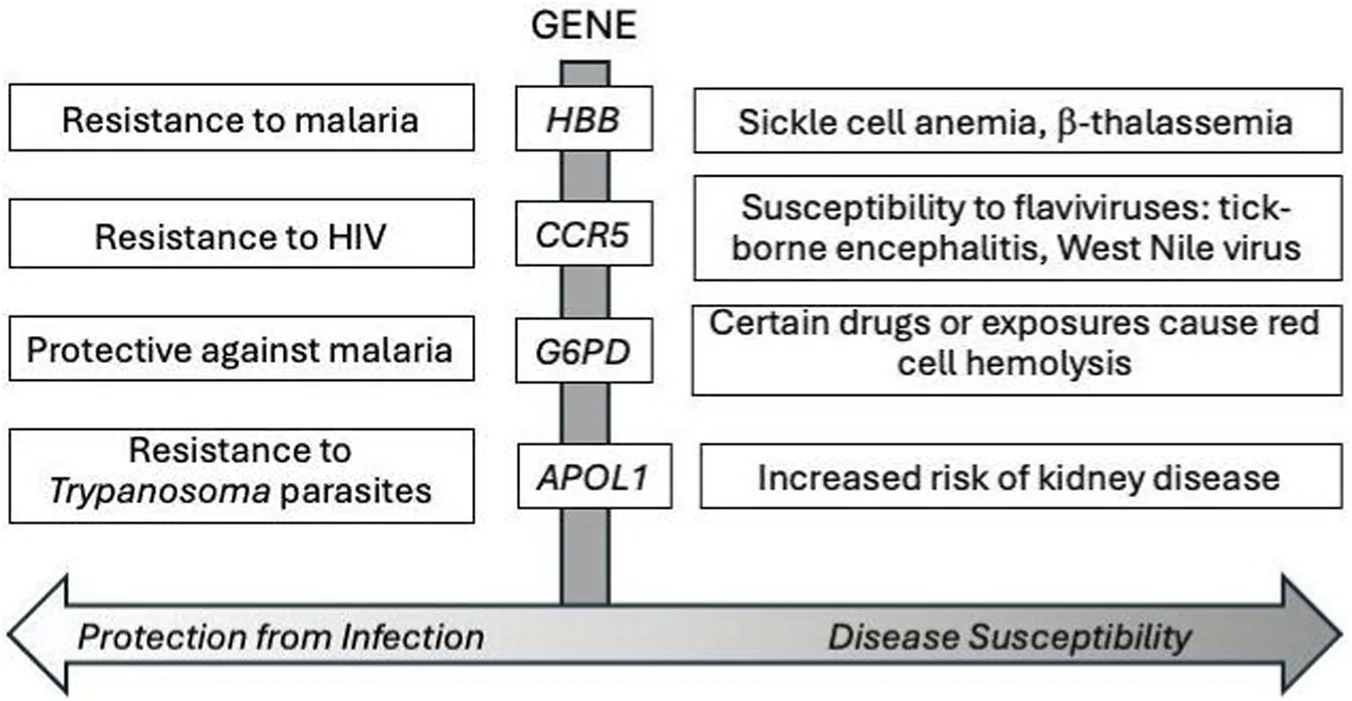
Examples of human genes with variants that provide protection from or susceptibility to infectious diseases, chronic diseases, and adverse pharmacogenomic interactions.

Pharmacogenomics can also contribute to optimizing infectious disease treatments through genotype-driven strategies to reduce adverse events and improve efficacy. For example, patients harboring the m.1555A>G variant in the mitochondrial 12S rRNA gene are at increased risk for aminoglycoside ototoxicity, making them vulnerable to hearing loss upon aminoglycoside exposure. While aminoglycosides are frequently used in pulmonary exacerbations in cystic fibrosis, screening for the m.1555A>G variant can guide clinicians toward alternative antibiotics or lower doses, sparing patients from permanent disability (McDermott et al., 2022). Similarly, plasma levels of dolutegravir, a key antiretroviral agent for treating HIV, are altered by variants in *UGT1A1*, influencing both its antiviral effectiveness and the risk of drug-related side effects (Spector et al., 2024). Identifying carriers of altered-function alleles of *UGT1A1* allows clinicians to adjust treatment regimens accordingly. Moreover, glucose-6-phosphate dehydrogenase (*G6PD*) deficiency, a common enzymopathy that makes red blood cells prone to hemolysis under oxidative stress, is protective and especially prevalent in malaria-endemic regions, but patients harboring certain genomic variants develop hemolytic anemia when exposed to drugs like primaquine for treating *Plasmodium vivax* malaria (Figure 1) (Gammal et al., 2023). While G6PD testing is available upon request in many healthcare settings, a broader adoption of enzymatic or genetic point-of-care testing, could help avert serious complications.

Although routine pharmacogenomic screening holds substantial promise, scaling it across diverse healthcare settings remains challenging. Additional research is required to define best practices for integrating pharmacogenomics into EHRs and reimbursement frameworks and to validate their cost-effectiveness. Single-gene assays deliver rapid insights in high-risk scenarios, but multi-gene panels that feed results seamlessly into EHRs may be more clinically useful and economical. Embedding these panels within EHRs and linking them to well-designed clinical decision-support systems would provide clear, actionable guidance for patient management.

Genomic variant frequencies differ markedly among ancestral populations, and clinically significant alleles may be confined to specific groups (Bastard et al., 2022). The lack of ancestral diversity in genomic datasets has been well-documented, particularly for genome-wide association studies, in which over 91% of the millions of persons included have been of European ancestry (Fitipaldi and Franks, 2023). Without genomic datasets that represent broad ancestral diversity and include robust pathogenicity annotations, individuals at risk for adverse genotype-driven outcomes remain undetected. Efforts to address this enormous data gap have included the All of Us Research Program (All of Us Research Program Genomics Investigators Collaborators, 2024), Global Biobank Meta-analysis Initiative (Zhou et al., 2022), and Human Heredity and Health in Africa (Sengupta et al., 2025). The value of these datasets is exemplified by the increased yield of associations from populations with high genetic diversity; for example, analysis of global genome-wide association data showed that 7% of associations were contributed by only 2.4% of persons of recent African ancestry (Jaffe et al., 2024). Clinically, these data gaps are manifested as reduced ability to classify variants as pathogenic/likely pathogenic or benign/likely benign, leaving higher proportions of VUSs in persons of non-European ancestry (Chen et al., 2023). Research initiatives must therefore prioritize enrollment from a wide spectrum of ancestries and ensure comprehensive curation of variant effects.

The development of multianalyte host-response biomarkers, incorporating genomic, transcriptomic, metabolomic, and proteomic markers (among others) shows promise for improving the diagnosis and treatment of infectious diseases (Buturovic et al., 2025; Ko and Tsalik, 2022). Several PCR-based platforms quantify immune markers that identify infections and guide therapy, and their deployment could support rapid, scalable, host-response-based testing to enable tailored interventions. Ultimately, integration of host genomics with population-level clinical data could shift infectious disease management from a reactive to an anticipatory paradigm, allowing clinicians to predict complications, select optimal therapies, and minimize adverse outcomes in a targeted, preemptive manner.

## Implementation

The substantial recent advances in understanding the genomics of host response to infections must now be translated into routine clinical practice to produce tangible benefits for patients and public health. While the scientific underpinnings of genomic medicine have progressed rapidly, real-world adoption remains inconsistent. Several implementation strategies merit consideration.

### Routine Genomic Sequencing Programs

The NIAID Centralized Sequencing Program is an example of how routine genomic sequencing can uncover actionable diagnoses (NIAID, 2025). All patients enrolled in NIAID protocols at the NIH Clinical Center are offered genome sequencing, with 95% acceptance. Results are interpreted by bioinformatics experts, who report primary diagnoses as well as secondary findings (Katz et al., 2020). These data have identified new and pathogenic variants, permitted modeling of polygenic risks, and contributed insights into structural genomic variants and somatic mosaicism. Patients receive genetic counseling, and information is available for clinical management. Such programs, however, require sustainable funding, specialized staff, and robust data-management pipelines. The success of the NIAID sequencing program demonstrates how this investment can speed diagnosis, target treatment, and reduce the financial and human costs incurred by ‘diagnostic odysseys” suffered by many patients whose underlying conditions remain obscure for months or years. Whether similar benefits can be realized in unselected populations of individuals without recognized immune or infectious phenotypes remains to be studied.

### EHR Integration and Clinical Decision Support

Even when genomic testing is performed, results often are trapped in private databases or embedded as static documents, limiting their global clinical utility. Full integration of genomic data into EHRs in structured, searchable formats would enable dynamic, algorithm-driven decision support, including genotype-specific contraindication alerts, dosing recommendations, and early screening reminders. To remain current, these data should be updated as new variants are classified. Artificial intelligence (AI) could enhance this system by capturing individual data and reporting associations of particular patient genotypes and phenotypes to flag actionable findings (Kingsmore and Cole, 2022).

One immediate strategy for identifying patients with inborn errors of immunity is to convert the Jeffrey Modell Foundation’s Ten Warning Signs (Warning Signs of Primary Immunodeficiency, 2025) into computable phenotypes (Rider et al., 2024). Retrospective analysis of these signs of primary immunodeficiency in over 150 million U.S. patient records showed that individuals with diagnosed IEIs were over tenfold as likely to exhibit two or more of these signs that have specific ICD-10 codes (e.g., bronchiectasis, splenomegaly, and certain pneumonias) (Rider et al., 2024). Digitizing warning signs within EHRs and applying AI-based analytics could enable continuous scanning for patterns consistent with immunodeficiencies, automatically triaging patients for further investigation and potentially streamlining insurance coverage decisions (Rider et al., 2024). A recent review reported high diagnostic accuracy, improved detection of pathogenic variants, and enhanced prediction of clinical outcomes for a variety of AI techniques (Taietti et al., 2025).

Clinical care could also benefit from adopting pathogen-genomic approaches pioneered in public health. Incorporating next-generation sequencing data on infectious agents into EHRs could accelerate pathogen identification, facilitate infection control, and guide antimicrobial therapy (particularly for drug-resistant strains) (Wilson et al., 2019; van Belkum et al., 2019; Snitkin et al., 2012). By embedding both host and pathogen genomic data into routine clinical workflows, healthcare systems could move from reactive to anticipatory management of infectious and immunological diseases.

### Reimbursement and Cost Effectiveness

Reimbursement can present barriers to implementation of genomic sequencing for infectious disease management. Payors often approve genomic tests only after conventional diagnostics have failed, thus delaying diagnosis and perpetuating a “failure-based” approach (Deverka et al., 2014; Krumm and Shirts, 2019). While insurers need evidence that genomic tests produce net savings or meaningful clinical improvements, large prospective trials or retrospective analyses that convincingly demonstrate such value will require multicenter, interdisciplinary collaboration. Professional societies and academic centers could collaborate to conduct well-structured studies showcasing the real-world advantages and potential cost savings of early genomic testing for IEIs and severe infectious diseases. Legislation mandating insurance coverage for biomarker testing could ensure that these tests are reimbursed. However, potential benefits of laws to expand access might be offset by overuse, which could diminish clinical utility and incur unnecessary costs (Lin et al., 2024).

### Ethics and Privacy

Unlike other personal health information genetic data are considered sensitive as they are uniquely identifiable, persistent across one’s lifetime, and potentially informative about biological relatives. Thus, every effort should be made to keep them confidential and secure, including secure transmission and handling among laboratories, databases, care providers, and patients. In addition, genetic information may profoundly impact an individual’s sense of self and perception of health. Pre- and post-test counseling and informed consent should explain both the powers and the limitations of available genetic tests.

## Discussion and directions

Advances in genomic knowledge and technologies hold great potential for improving infectious disease prevention and management, but they have outpaced the ability of health systems to implement these new tools effectively. While early detection of IEIs has already transformed outcomes for many patients, a vast untapped potential remains for expanding genotype-first approaches, refining genomic-variant interpretation pipelines, and integrating genomic decision support into care for infectious diseases and immune disorders.

Moving forward, the responsibility for progress rests with the entire genomic and infectious disease communities. Innovative programs that generate robust proof-of-concept data—especially regarding cost-effectiveness—can strengthen the case for policy reforms that support coverage for genomic testing and harmonize clinical guidance to deploy these tests in an effective and timely way to inform diagnosis and care. Additionally, partnerships among government agencies, academic consortia, non-profit advocacy groups, and private sector leaders must create a coordinated strategy for data sharing, standardized genomic variant annotation, and equitable access to genomic testing. Incorporating selected, impactful, IEI-related genomic variants, or even genome sequencing, into newborn screening programs can be considered in the future but will require substantially more evidence of cost-effectiveness as well as fuller understanding of appropriate referral and follow-up actions before becoming widespread.

New technologies, such as long-read DNA sequencing, hold promise for resolving ambiguities to improve interpretation of variants associated with several IEIs. Higher-redundancy sequencing will allow more somatic genomic variants to be identified, opening new windows into acquired causes of susceptibility. Further, the microbial data captured in metagenomic sequencing of clinical samples has only begun to be tapped. Microbial data can assist with diagnosis and inform treatment. Collaboration among major informatics consortia will be needed to standardize data formats and classification schemas, ensuring that novel genomic variants are quickly integrated into public databases. Finally, user-friendly workflows for diagnosing IEIs, which include illustrated guides for navigating ClinGen and ClinVar, would help frontline clinicians harness the full promise of genomically informed care.

Early and accurate genomic diagnoses can streamline treatments, avert serious complications, and improve patients’ health and quality of life. Further successes require resolving implementation barriers, including (i) developing robust genomic variant curation across all ancestral origins; (ii) integrating genomic data and updated interpretation into the EHR; (iii) working out sustainable reimbursement policies; and (iv) providing front-line clinicians with accessible AI-driven tools. By embracing cross-sector collaborations between the federal government, academia, and industry, the field can progress toward a model in which genotype-first diagnostics, targeted pharmacotherapies, and adapted infection control measures become the global standard, ultimately safeguarding patient health and improving patient outcomes more efficiently than ever before.
